# Human telomerase reverse transcriptase binds to a pre-organized hTR *in vivo* exposing its template

**DOI:** 10.1093/nar/gkv1065

**Published:** 2015-10-19

**Authors:** Georgeta Zemora, Stefan Handl, Christina Waldsich

**Affiliations:** Department of Biochemistry and Cell Biology, Max F. Perutz Laboratories, University of Vienna, Dr Bohrgasse 9/5, A-1030 Vienna, Austria

## Abstract

Telomerase is a specialized reverse transcriptase that is responsible for telomere length maintenance. As in other organisms, the minimal components required for an active human telomerase are the template-providing telomerase RNA (hTR) and the enzymatic entity telomerase reverse transcriptase (hTERT). Here, we explored the structure of hTR and the hTERT-induced conformational changes within hTR in living cells. By employing an *in vivo* DMS chemical probing technique, we showed that the pseudoknot and associated triple helical scaffold form stably *in vivo* independently of hTERT. In fact, the dimethyl-sulfate (DMS) modification pattern suggests that hTR alone is capable of adopting a conformation that is suited to interact with hTERT. However, in the absence of hTERT the template region of hTR is only weakly accessible to DMS-modifications. The predominant change after binding of hTERT to hTR is the exposure of the template region.

## INTRODUCTION

Telomerase is a ribonucleoprotein (RNP) complex that adds tandem repeats at the ends of the linear chromosome to counteract for the loss of sequence due to the DNA end replication problem (1,2). The repeats are organized as telomeres and they form the protective end-caps of eukaryotic chromosomes. Telomerase has become the focus of medical research because telomerase is upregulated in the vast majority of cancers (3) and mutations in the telomerase components have been associated with a large spectrum of premature aging disease (4).

**Figure 1.**
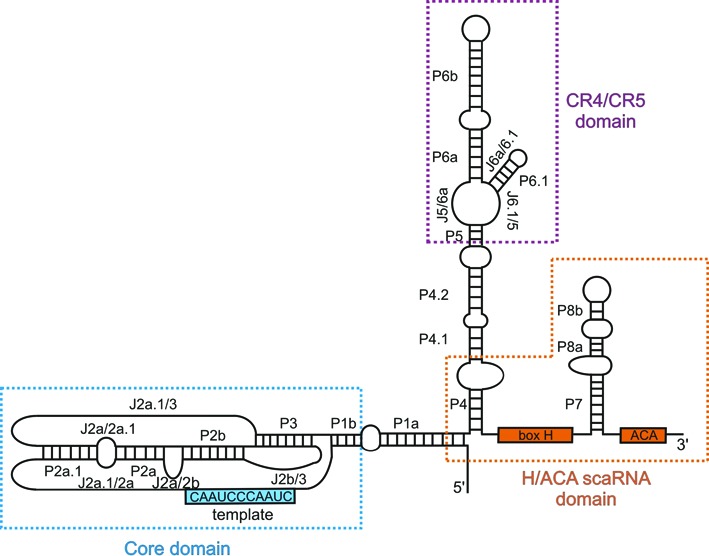
The secondary structure organization of hTR. The three major structural domains are boxed: the core domain (pseudoknot/template domain) in blue, the CR4/CR5 domain in purple and the H/ACA scaRNA domain in orange. The template is indicated as blue rectangle containing the sequence and the conserved boxes H and ACA are displayed as orange rectangles.

The telomerase core components important for the telomeric repeat synthesis are the telomerase reverse transcriptase (TERT) and its integral telomerase RNA component (TR) (5). Although these two components are sufficient to reconstitute telomerase activity *in vitro* (6), the telomerase holoenzyme *in vivo* contains additional proteins required for telomerase biogenesis, stability and localization (7). These are the H/ACA-binding proteins and TCAB1 associated with scaRNAs (7). While TERT is highly conserved across eukaryotes, TR is highly divergent, both in size and sequence (8). The mature human telomerase RNA (hTR) is a 451 nt long transcript and phylogenetic comparison of vertebrate TR sequences identified eight conserved regions (CRs), which are part of three structural domains (Figure 1): (i) pseudoknot/template (CR1–CR3) domain (core domain); (ii) CR4/CR5 domain and (iii) H/ACA scaRNA domain (CR6–CR8) (8,9). Structurally, the 5′ region comprising the pseudoknot/template domain and the CR4/CR5 domain are important for catalytic activity of the telomerase and the former provides the template for the telomere repeat synthesis (10), while the 3′ region harbouring the boxes H and ACA and CR7 (scaRNA domain) is important for hTR biogenesis, stability and localization (8,11–13). Regarding the catalytic subunit, it has been reported that hTR makes two independent contacts with hTERT: via its pseudoknot/template domain and through the CR4/CR5 domain (14,15).

The current secondary structure model of hTR (Figure 1) is based on phylogenetic comparison and co-variation data as well as mutational analysis (8,16). Parts of the conserved hTR regions have been structurally characterized by NMR spectroscopy (17–23). For example, the solution structure of the minimal P2b/3 pseudoknot (17,18) revealed the formation of a triple helix that contributes to catalysis (24). Recently, high-resolution structures of the CR4/CR5 domain of the teleost fish medaka (*O. latipes*) have provided a detailed view on the structural organization of this domain (25,26). While significant progress has been made in understanding structural aspects of hTR (9,13,17–23), the conformation of the full-length hTR remains elusive, both *in vitro* and *in vivo*.

To shed light on the structure of full-length hTR transcript and on its assembly with hTERT, we mapped the RNA's conformation in living cells employing an *in vivo* chemical probing technique. The dimethyl-sulfate (DMS) modification pattern revealed that hTR mostly forms the predicted secondary structure and allows drawing parallels with the high-resolution structures determined for individual, isolated hTR fragments. Foremost, our results indicate that the pseudoknot and associated base triples form *in vivo* and hTR holds several tertiary interactions to be discovered. Comparing the modification data in the absence and presence of hTERT indicated that there are local structural alterations within the CR4/CR5 domain, the extended pseudoknot and the template region, which becomes more exposed in the presence of hTERT. In contrast, the structure of the pseudoknot itself and of elements that are important for hTR maturation and accumulation appears to be similar regardless of the hTERT presence. This suggests that no major overall structural rearrangements take place upon binding of hTERT. Moreover, we identified several pseudouridines beyond those previously reported within hTR (22), which are likely to play a role in hTR structure and function.

## MATERIALS AND METHODS

### Plasmid constructs, hTR mutagenesis and transient transfection

Plasmids used for the transient expression of hTERT (pcDNA6–hTERT) and hTR (pBS-U1-hTR) in HEK293 cells were a kind gift from Prof. Dr Joachim Lingner (EPFL, Switzerland) and they were previously described (27). The hTR variants were based on pBS-U1-hTR vector and were created by the FastCloning method (28). HEK293 cells were transfected with 4 μg plasmid and 7.5 μl Nanofectin (PAA) in 6 well-plates following the manufacturer's instructions. The plasmid ratio for hTERT and hTR expression was 5:1 (3.33 μg pcDNA6-hTERT and 0.66 μg pBSU1-hTR). After 24 h the transfected cells were transferred to a 10 cm ødish. 72 h post transfection, cells were detached and used for one of the following: *in vivo* DMS modification, preparation of telomerase extracts or for detecting pseudouridines in hTR.

### *In vivo* DMS modification and total RNA extraction

*In vivo* DMS modification of HEK293 cells followed by total RNA extraction was recently described in detail (29).

### Reverse transcription

Five sequence-specific DNA primers were used for reverse transcription to map the modification sites within hTR: hTR_149: 5′ GTTTGCTCTAGAATGAACGGTG 3′, hTR_178: 5′ GAACGGGCCAGCAGCTGACA 3′, hTR_238: 5′ GCCTCCAGGCGGGGTTCG 3′, hTR_404: 5′ GTCCCACAGCTCAGGGAATC 3′ and hTR_433: 5′ GCATGTGTGAGCCGAGTCC 3′. Using 5′ ^32^P-end-labeled primers reverse transcription was performed as reported in (29). The cDNAs were resolved on an 8% denaturing polyacrylamide gel (29). The dried gel was exposed to a phosphorimager screen for 24 h, after which the screen was scanned using a Typhoon (GE Healthcare).

### Quantification of DMS modifications

The band intensities were quantified using ImageQuant V7 (GE Healthcare). After lane/band selection as well as background correction, the output number of the band intensities was exported to Excel (Microsoft). After normalization, the changes in the intensity of DMS modification were calculated from the ratio of counts in the presence and absence of hTERT. We set a threshold value of 1.5 to determine whether differences were significant or not; this cut-off value has been previously used for similar studies (30–32). Modification intensities that are ≥1.5-fold higher in the presence of hTERT were considered as an enhancement, while those that were ≥1.5-fold smaller in the presence of hTERT were referred to as a protection. Average values were calculated from at least three independent experiments.

### Direct telomerase assay

Telomerase extracts were prepared and the direct telomerase assay (DTA) was performed as described by Cristofari and Lingner, 2006 (27) and Cristofari *et al*., 2007 (33), respectively, with minor modifications. 20 μl reactions containing 20 μg of total protein extract, 50 mM Tris-HCl pH 8.0, 50 mM KCl, 1 mM MgCl_2_, 1 mM spermidine, 5 mM β-mercaptoethanol, 0.5 mM dATP, 0.5 mM dTTP, 2 μM dGTP, 20 μCi [α-^32^P] dGTP (3000 Ci/mmol) and 1 μM telomeric primer (5′ (T_2_AG_3_)_3_ 3′) were incubated at 30°C for 1 h. RNA was degraded with 5 μl RNase (10 μg/μl) at 37°C for 10 min. Proteins were digested with 15 μl of Proteinase K (20 mg/ml, AppliChem) at 37°C for 30 min. After adding trace amounts of a ^32^P-labeled 100-mer DNA oligo the samples were precipitated and subsequently half of each sample was resolved on an 8% denaturing polyacrylamide gel. After exposing the dried gel to a phosphorimager screen for 24 h, the screen was scanned using a Typhoon (GE Healthcare). Following the analysis with ImageQuant V7 (GE Healthcare), telomerase activity and processivity were calculated as described (34).

### Mapping pseudouridines in hTR

To map the possible pseudouridines in hTR, we used a previously published protocol (35) with minor modifications. 20 μl total RNA (1.5 μg/μl) were mixed with 80 μl BEU buffer (7 M urea, 4 mM EDTA pH 8.0, 50 mM bicine pH 8.5; the final pH should be around 8.9–9) and 20 μl 1 M CMCT (Sigma-Aldrich). A sample that was not treated with CMCT was prepared as negative control. The reactions were incubated at 37°C for 10 min to allow CMCT modification of G, U and Ψ residues. After ethanol precipitation, the RNA pellet was dissolved in 50 μl sodium carbonate buffer pH 10.4 (50 mM sodium carbonate pH 10.4, 2 mM EDTA pH 8.0) and incubated at 37°C for 4 h to allow the removal of CMCT from G and U residues. Following ethanol precipitation, the RNA pellet was dissolved in 10 μl 25 mM borate buffer pH 8.0. The Ψ residues were mapped by reverse transcription and the cDNA pool was resolved on an 8% denaturing polyacrylamide gel. After exposure of the dried gel to a phosphorimager screen for 24 h, the screen was scanned using a Typhoon (GE Healthcare) and the gels were analysed with ImageQuant V7 (GE Healthcare).

## RESULTS

### Experimental setup

**Figure 2.**
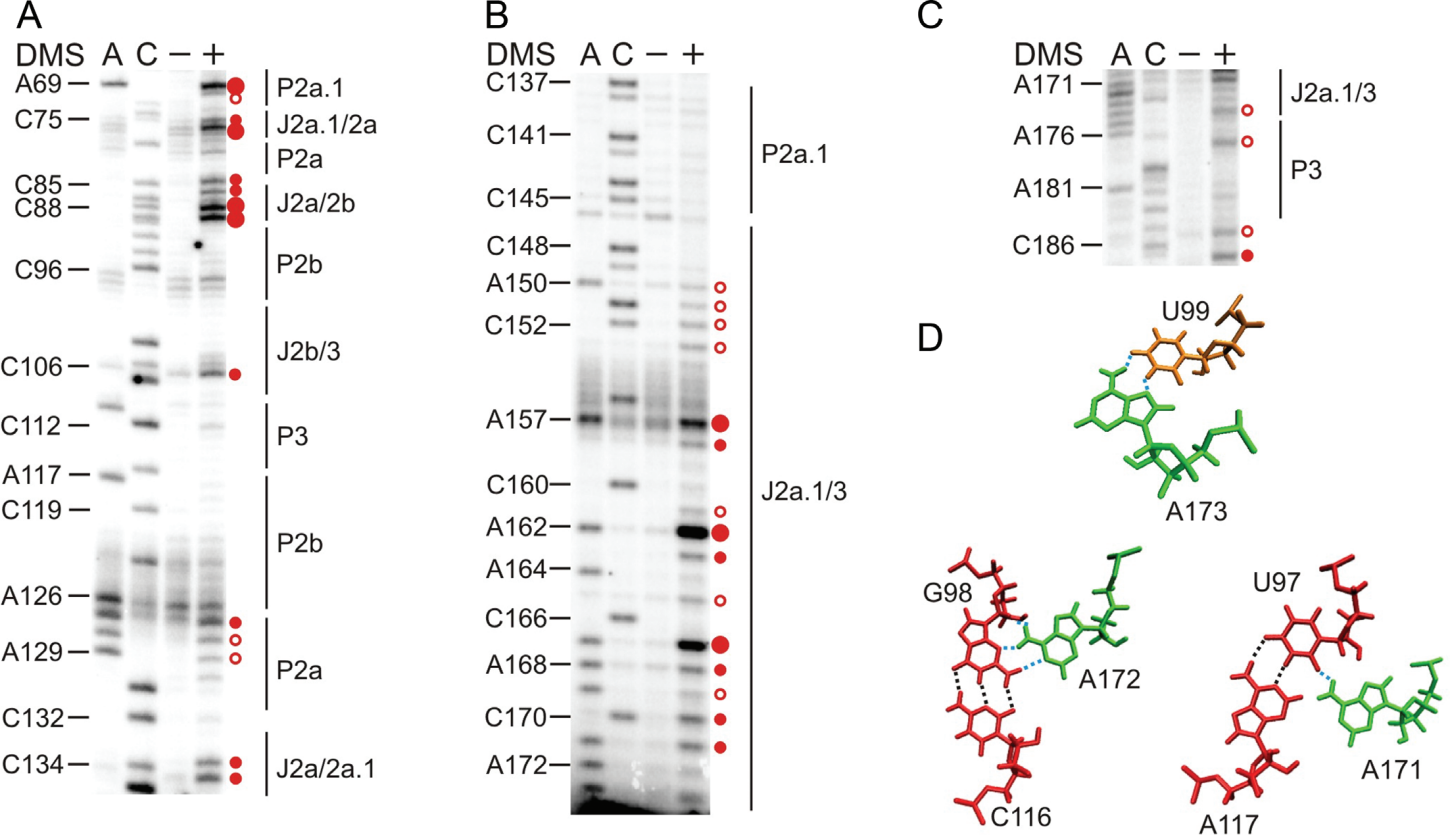
The P2b/P3 pseudoknot and its peripheral extensions are formed *in vivo*. Representative primer extension gels showing the *in vivo* DMS modification pattern of: (**A**) stems P2a.1 (5′ strand), P2a, P2b and their respective junctions; (**B**) P2a.1 (3′ strand) and J2a.1/3; (**C**) P3 (3′ strand). A and C denote sequencing lanes, generated from the RNA that was not treated with DMS. The – lane shows the natural stops encountered during reverse transcribing unmodified hTR into cDNA. The + lane displays the *in vivo* DMS modification pattern of hTR. The DMS-induced stops (red circles) are revealed by comparing the – and + lanes and excluding the natural stops, thereby revealing the accessible A (N1) or C (N3) residues within the hTR. The size of the circle correlates with the relative modification intensity of individual bases. Red open circles indicate residues with weak reactivity to DMS, while red filled circles represent strongly (large circles) or moderately (small circles) modified nucleotides, respectively. The DMS modifications were plotted onto the secondary structure map of hTR (Figure 3). (**D**) Tertiary contacts identified in the solution structure of the hTR P2b/P3 pseudoknot (17). H-bonds with the two minor groove base triples and the Hoogsteen A•U base pair are indicated with blue dotted lines, while those of the canonical Watson–Crick interaction are labelled with black dotted lines.

**Figure 3.**
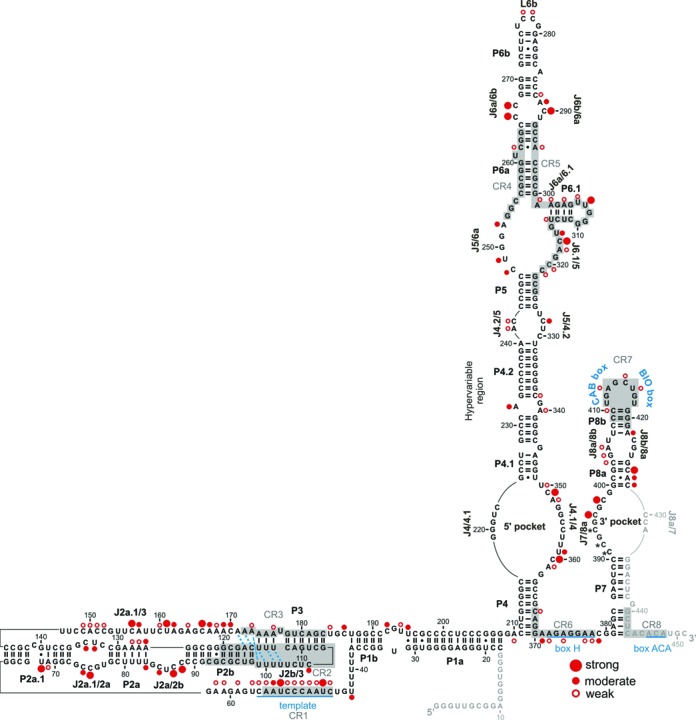
The intracellular structure of hTR. The map summarizes the A, C and U residues of hTR that are accessible to DMS in the presence of hTERT, as indicated with red circles. Open circles indicate residues with weak reactivity to DMS, while filled circles mark strongly (large circles) or moderately (small circles) modified nucleotides. In the pseudoknot the base triples (U100•U115-A174, U101•U114-A175, U102•U113-A176, U97-A117•A171, G98-C116•A172) are indicated with a blue dotted line, while the U99•A173 pair is marked with a blue dashed line. Residues coloured in light grey (nts 1-17 and 430–450) could not be mapped by reverse transcription, as the very 3′ end served as primer binding site and the very 5′ end (C8, A10) were not well resolved on the gel. Average values were calculated from at least three independent experiments.

**Figure 4.**
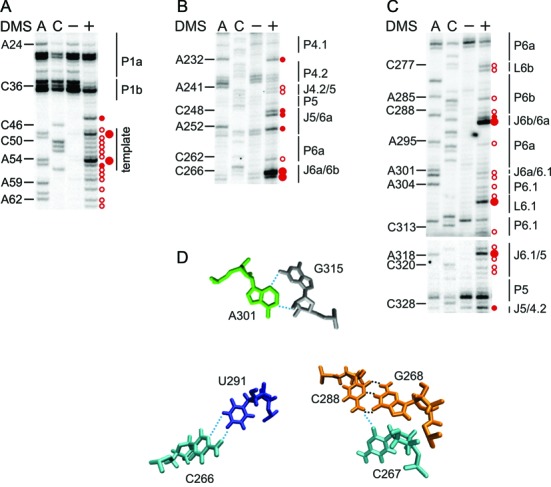
The structure of the template region and CR4/CR5 domain is well defined *in vivo*. Representative primer extension gels showing the *in vivo* DMS modification pattern of the (**A**) template region, (**B**) 5′ and (**C**) 3′ part of the CR4/CR5 domain. Lanes and the symbol code are designated as in Figure 2. (**D**) Tertiary contacts observed in the crystal structure of the medaka CR4/CR5 domain in complex with TRBD (26) and in the solution structure of the P6a/P6b element (21) are shown. In the upper panel the A301•G315 non-canonical base pair is shown, while the C266•U291 pair and the C267•G268-C288 base triple are found in the lower panel. All nucleotide numbering is according to hTR.

**Figure 5.**
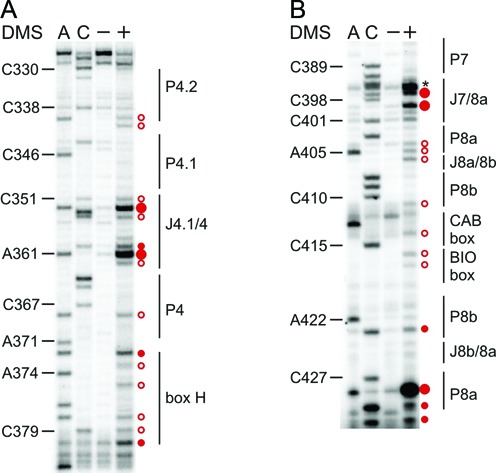
The intracellular fold of the H/ACA scaRNA domain. Representative primer extension gels showing the *in vivo* DMS modification pattern of: (**A**) the hypervariable region together with the 5′ pocket embedding stems P4.1 and P4; and (**B**) the 3′ H/ACA hairpin harbouring the 3′ pocket and the CAB and BIO boxes. Lanes and the symbol code are designated as in Figure 2.

**Figure 6.**
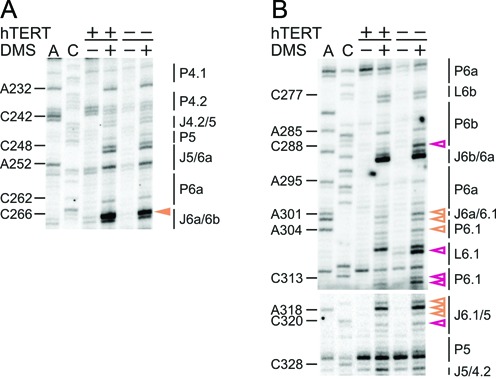
hTERT influences the structure of the CR4/CR5 three-way junction. Representative gels showing the modification intensity of the nucleotides in the CR4/CR5 domain of hTR, in the presence (hTERT +) and absence (hTERT−) of hTERT: (**A**) 5′ strand and (**B**) 3′ strand of the CR4/CR5 element. The arrowheads indicate residues, the accessibility of which changed is in the presence of hTERT. Intensity values that are 1.5-fold higher in the presence of hTERT are considered as enhancement (filled orange arrowheads) and those 1.5-fold or 2-fold lower were considered as protections (open orange and open magenta arrowheads, respectively). These values were derived from normalized plots (Supplementary Figures S4 and S5). Lanes are designated as in Figure 2. Comparing lanes 4 and 6 reveals the altered DMS modification pattern and thus structural changes in hTR upon binding of hTERT.

**Figure 7.**
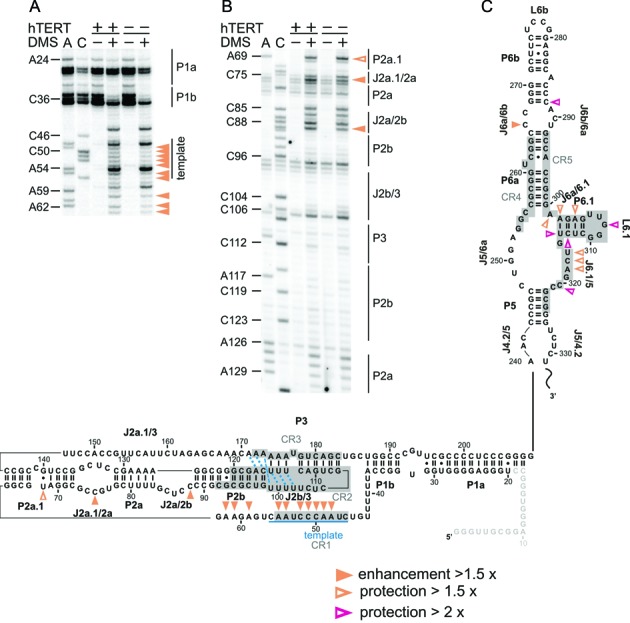
hTERT-induced conformational changes within the hTR *in vivo*. Representative gels showing the modification intensity of the nucleotides in the (**A**) template region and (**B**) pseudoknot of hTR in the presence and absence of hTERT. Lanes and the symbol code are designated as in Figure 6. (**C**) Differential map: residues, the modification intensity of which changes upon hTERT binding are indicated. The orange filled arrows mark an increase in accessibility (enhancement) in the presence of hTERT; open arrows represent bases with reduced accessibility (protection) in the presence of hTERT. The A, C and U residues whose modification remains unaltered (i.e. equally modified in the presence and absence of hTERT) are not highlighted. This map is based on normalized plots (Supplementary Figures S4 and S5). Average values were calculated from at least three independent experiments.

In order to determine the intracellular structure of hTR, we performed *in vivo* structural probing with DMS (29). DMS readily penetrates the cells, thereby modifying the N1 of adenines and the N3 of cytosines, if these nitrogens are not involved in H-bonding or protected by a protein or a ligand. Since the endogenous hTR accumulates at very low levels in HEK293 cells, mapping of hTR via DMS chemical probing was compromised by a poor signal-to-noise ratio and often by mispriming due to the GC-rich content of hTR (data not shown). To overcome this, we overexpressed the two key components of telomerase, hTERT and hTR, in HEK293 cells. The plasmids encoding hTERT and hTR were cotransfected in HEK293 cells at a 5:1 ratio, thus maintaining hTR at a low level but sufficient to map the intracellular hTR structure (Figures 2–5 and Supplementary Figure S2) and to infer hTERT-induced conformational changes in hTR (Figures 6 and 7, Supplementary Figures S4–S7). To ensure that the telomerase RNP complex is indeed formed and functional in our system, the *in vivo* reconstituted telomerase complex was assayed *in vitro* for its activity (Supplementary Figure S1). As previously reported the recombinant telomerase was active only when both hTERT and hTR were co-transfected (27). As a functional telomerase is only detectable when both recombinant hTERT and hTR are present, this excludes any significant contribution of endogenous hTR or hTERT. This encouraged us to use these components as a model for determining the intracellular structure of hTR and the hTERT-induced conformational changes within hTR. 72 h post transfection, HEK293 cells were treated with DMS, and then the total RNA was extracted from cells. The sites of DMS modifications were detected by reverse transcription and plotted onto the secondary structure of hTR (Figure 3). Depending on the band intensity, the modifications of As and Cs were assigned as strong, moderate or weak. Since U and G residues are occasionally reactive to DMS at their N3 (U) or N1 (G) (36), if their local environment stabilizes the formation of keto-enol tautomers of respective Us and Gs (37), these have been included in the analysis as well. At first we determined the hTR fold in the context of hTERT, as we would expect to find the RNP complex assembled inside the cell (Figures 2–5).

### The pseudoknot is formed stably *in vivo*

The P2b/P3 pseudoknot contains numerous highly conserved nucleotides (CR2/CR3). Correct folding of the pseudoknot and associated tertiary interactions have been shown to be essential for telomerase function (18,24,38,39). As such, we aimed at exploring the structure of the P2b/P3 pseudoknot and its extensions (P2a, P2a.1) within the hTR in the presence of hTERT in living cells.

The A and C residues within stems P2b and P3 (except for A176) show no reactivity to DMS (Figures 2 and 3), suggesting completely paired helices. A176, which forms a Watson–Crick base pair with U113, is weakly modified (Figures 2C and 3). Importantly, A176, specifically its 2′OH group, has been found to be important for telomerase catalysis, therefore requiring backbone flexibility (24), which might explain the weak accessibility to DMS. As observed by NMR (17,18), the P2b/P3 pseudoknot is further stabilized by a triple helix. Minor groove base triples are formed between P2b and J2a.1/3, while major groove base triples occur between P3 and J2b/3. Specifically, the last base pair in P2b, G98-C116, interacts with A172 via its Watson–Crick face (N1 and N6), while the penultimate base pair U97-A117 in P2b contacts A171, forming a H-bond between N6 of A171 and C2 carbonyl of U97 (17,18) (Figure 2D). In fact, both A171 and A172 are not accessible to DMS *in vivo* in the context of the full-length hTR (Figure 2C), suggesting that the base triples are formed. C170 stacks on A171, but does not appear to hydrogen bond with the stem P2b (18). In fact, C170 is moderately modified by DMS (Figure 2B), indicating that its N3 is not involved in a tertiary interaction. Given that the major groove base triples are observed *in vivo*, it is well possible that the minor groove base triples formed between U–A base pairs (U113–A176, U114–A175, U115–A174) in P3 and U100–U103 in J2b/3 take place *in vivo* as well. Adjacent nucleotides C104 and C106 are not or moderately modified, respectively (Figure 2A). At the junction between P2b and P3, the first nucleotide in J2b/3 (U99) interacts with the last nucleotide of J2a.1/3 (A173) to form a Hoogsteen A·U base pair (Figure 2D), which is critical for pseudoknot architecture and telomerase activity (17). In line with this non-canonical base pair, A173 is accessible to DMS, but the modification is weak. This may indicate that A173 has reduced solvent accessibility due to the intricate H-bonding network within the P2b/P3 pseudoknot. Notably, for the remainder of the long junction connecting P2a.1 with P3 there is no structural information available. The *in vivo* DMS modification of J2a.1/3 revealed only three strongly modified residues, U156, U161 and C166, respectively, while most of the J2a.1/3 nucleotides are moderately (A157, A162, A167, A169 and C170) or weakly (C149, A150, C151, C152, C160, A164 and A173) modified and C148 is not modified (Figure 2B). Overall, the DMS modification pattern of the residues in P2b/3 indicates that the pseudoknot and triple helix are formed stably *in vivo*.

The extension of the pseudoknot comprising stems P2a and P2a.1, which are separated by an internal loop and junction J2a/2b, do not show a high degree of sequence conservation. However, the integrity of all helices is required for creating a stable pseudoknot fold (40). The *in vivo* DMS modification pattern suggests that the mammalian-specific stem P2a.1 is formed, as most of the nucleotides are not modified except for U68 (Figure 2A). This highly conserved residue, which has been predicted to form a G·U wobble base pair with G140, is strongly modified, indicating that this base pair may not form or adopts a different pairing geometry that does not involve the N3 atom of U68. Since A69 is also weakly modified (Figure 2A), this might favour the idea of U68 being unpaired and in turn loosening the A69–U141 base pair. Mutational analysis indicated that stem P2a.1 is 2 base pairs longer (A62-U147, G63-U146) than previously suggested based on phylogeny (40). If this is the case, the weak DMS modification at A62 might come as a consequence of these two base pairs being positioned at the end of the P2a.1 helix. In the internal loop that separates P2a.1 and P2a, only C75 is strongly modified, while the rest of the residues are either moderately (C74, U133, C134) or not modified (C132, Figure 2A). As for stem P2a, we observed that its terminal Watson–Crick base pair U83–A126 is not formed, as both residues are moderately methylated (Figure 2A). Moreover, the adenines of the adjacent base pairs are also weakly modified by DMS. As observed by NMR (19), the conformation of J2a/2b introduces a large bend between stems P2a and P2b across the major grove and, in line with our data, this rare structural motif has been reported to weaken the stability of neighbouring base pairs. In J2a/2b the residues C85, C87, C88 are moderately and strongly accessible to DMS, respectively, (Figure 2A) suggesting that this pyrimidine-rich loop is formed in *in vivo*.

### The template region is rather buried within the telomerase RNP

Within the template region all As and Cs were sensitive to DMS, but exhibit only low (C46, A48, A49, C50, C51, C52, C55 and C56) or moderate (A54) reactivity (Figure 4A). Also, adjacent adenines A59, A61 and A62 are only weakly methylated by DMS. Surprisingly, four uracils were found modified; U47 and U53, which are located in the template, are strongly reactive to DMS, while flanking residues U43 and U57 show moderate and weak reactivity, respectively (Figure 4A). This implies that the junction J1b/2a.1 harbouring the template adopts a specific geometry within the hTR–hTERT complex, resulting in limited reactivity and/or accessibility of the template to DMS. As such, the template might be rather buried within the telomerase RNP, but retaining its ability to interact with telomeres at the same time.

An essential element for proper telomere elongation is the template boundary element (TBE). Earlier studies indicated that in hTR the TBE is ensured by the P1b stem together with the length of the flanking linker to the template (41). More recent experiments have shown that the P1b stem is not sufficient to prevent template boundary bypass and in addition a sequence-based template mechanism is required for the strict definition of the template boundary (42). As residues (C190, C191) in P1b are not accessible to DMS (C35, C36 cannot be assessed due to natural stops of the reverse transcriptase), this suggests that the P1b is formed *in vivo* (Supplementary Figure S2). Along this line, the adjacent stem P1a is established as well, since residues therein (A24, C203–C205) are not modified (again numerous RT stops were observed in this region; Figure 4A and Supplementary Figure S2).

### The architecture of the CR4/CR5 domain

The functionally essential elements of the CR4/CR5 domain include the conserved residues of the P5-P6a-P6.1 three-way junction and the internal loop between P6a and P6b (14). Recently, the crystal structure of the medaka TRBD domain in complex with CR4/CR5 domain has been solved (26). In complex with TRBD the CR4/CR5 domain adopts an L-shaped three-way junction conformation, in which P5 and P6 coaxially stack, while P6.1 branches from the pseudo-continuous helix (26).

The *in vivo* DMS modification pattern suggests that helices of the three-way junction are properly formed, as most residues were not reactive to DMS (Figures 3 and 4B,C). In stem P6a we only observed weak modifications at C262 and A295 (Figure 4B,C). Based on phylogeny a C262•A295 base pair next to a U261 bulge was predicted (8) (Figure 3), while a U261–A295 base pair with a C262 bulge was observed in the NMR structure (21). If C262 is bulged out, a strong modification of this residue could be expected. Instead, the weak accessibility of C262 and A295 might point to a non-canonical C262•A295 base pair. Mutations that would disrupt the phylogenetically predicted C262•A295 or NMR derived U261–A295 base pairs did not significantly reduce telomerase activity nor did they affect the repeat addition processivity (RAP; Supplementary Figure S3). This might not exclude the possibility of a base triplet at these positions in the full-length hTR.

In case of the highly conserved stem-loop P6.1, A302, A304 and U314 showed weak DMS reactivity (Figure 4C). Interestingly, U306, 307 and U316 have previously been described as pseudouridines (22) and these nucleotides are modified by DMS as well (Figure 4C). In J6.1/5 C317 is strongly modified, while all other As and Cs are only weakly accessible to DMS. In the J5/6a, only the conserved C255 is not methylated by DMS, while all the other residues (C248, U249 and A252) are moderately methylated (Figure 4B).

In the crystal structure of the CR4/CR5–TRBD complex from medaka (26), A199 (A301 in hTR) makes three H-bonding interactions, one with TRBD and two with G213 (G315 in hTR; Figure 4D). This non-canonical A•G interaction stabilizes the bent backbone of the RNA and makes a sharp turn between stems P6 and P6.1 (26). As A301 in J6a/6.1 only displays a weak modification (Figure 4C), this implies that the non-canonical A•G interaction, in which the N1 and N3 of A199 contact the 2′OH and exocyclic amine of G213, respectively (26), forms in most hTR molecules as well. In support to this, mutants disrupting this non-canonical interaction significantly reduced telomerase activity (Supplementary Figure S3).

In the internal loop that separates P6a from the P6b, residues C266, C267 and C290 are strongly modified, while A289 is only moderately modified (Figure 4B,C). This is in good agreement with the finding that this internal loop has an unusual solvent-accessible opening (9,21). In fact, C266 was proposed to pair with U291 using its exocyclic amino group and N3 atom (21) (Figure 4D). In light of its strong modification it is unlikely that such a contact forms *in vivo*. The intense methylation of C267 correlates with the observation that its O2 atom forms a base triple with C288, which pairs with G268 (21), leaving its N3 accessible for modification (Figure 4D). The P6b seems to have a well-defined structure, and only the bulge A285 and the Cs in L6b (C277 and C278) are weakly accessible to DMS (Figure 4C).

### Assessing the conformation of the H/ACA scaRNA domain

In the phylogenetic analysis (8), the junctions J4.2/5 and J5/4.2 separate the CR4/CR5 domain from the hypervariable region. Residues in these junctions are weakly (A241 and C242) or moderately (C328) accessible to DMS, while C330 is protected from modification (Figures 4B,C and 5A). As for the hypervariable region and the helices embedding the 5′pocket, the DMS modification pattern indicates that P4, P4.1, P4.2 form stable stems *in vivo*, as the respective As and Cs are not modified by DMS (Figures 4B and 5A). In the 5′pocket (J4/4.1 and J4.1/4) residues are either strongly (C351 and C360) or moderately modified (U359) by DMS, while U350, A352 and A361 are weakly reactive to DMS and C223, C355 and C356 are protected from modification (Figure 5A and Supplementary Figure S2). Of the bulged nucleotides between P4.1 and P4.2, A232 is moderately modified (Figure 4B), while A340 is only weakly modified (Figure 5A). In light of the modification of residues in these asymmetric internal loops, it can be assumed that these form either intra-loop base pairings or engage in long-range interactions.

As for P7 and 3′ pocket, it is only possible to assess their conformation from the 5′part, as the 3' strand is part of the primer-binding site for reverse transcription. None of the nucleotides in P7 (5′ strand) show any reactivity to DMS, suggesting that P7 is formed (Figure 5B). In the 3′ pocket (J7/8a) C396 and C398 are strongly modified, while C391, C392 and C394 could not be assessed, because these positions are not well resolved on the denaturing PAGE and migrate as a single, very intense band, potentially indicating that these residues are also accessible to DMS (Figure 5B). In contrast to P7, all As and Cs in P8a are accessible to DMS, except for C401. While C427 is strongly reactive to DMS, A428 and C429 are moderately modified and C403 displays a weak modification (Figure 5B). Thus, this modification pattern implies that P8a, which is predicted to consist of three Watson–Crick and a non-canonical C•A base pair, does not form *in vivo*, potentially increasing the size of the 3′ pocket. Alternatively, a different base pairing geometry may occur that allows the N1 of A and the N3 of C to be methylated by DMS. The residues of the internal loop that separates stems P8a and P8b are either weakly (G404, A405) or not modified (C423, Figure 5B). As for stem-loop P8b, only residues involved in the formation of the two helix-enclosing base pairs (C410 and A422) are accessible to DMS (Figure 5B). Residues in the conserved loop L8b containing the CAB and BIO boxes show weak accessibility to DMS (A413, C415, U416; Figure 5B), suggesting their interaction with proteins required for hTR biogenesis and accumulation (7,43). Similarly, the adenines in box H, which together with box ACA are required for interaction with the H/ACA-binding proteins (7), show only weak accessibility to DMS (Figure 5A), while flanking nucleotides (A371, C379) are moderately modified by DMS. As the ACA motif is located at the very 3′end of hTR, it was impossible to map these residues by reverse transcription.

### hTERT induces discrete structural changes *in vivo*

Using *in vivo* DMS probing we aimed to determine how hTERT influences the conformation of hTR upon complex assembly. As such, we mapped the hTR structure also in the absence of hTERT, by transfecting HEK293 cells only with the plasmid encoding hTR. As telomerase activity could not be detected in the absence of recombinant hTERT (Supplementary Figure S1), we reasoned that the endogenous hTERT, the levels of which cannot be detected by Western blotting (27), is unlikely to significantly influence folding of the recombinant hTR. To discern the influence of hTERT on the hTR fold, we compared the *in vivo* DMS pattern of hTR in the presence and absence of hTERT (Figures 6 and 7).

Upon binding of hTERT to hTR discrete conformational changes were only observed in the CR4/CR5 domain, the template region and in the extended pseudoknot (Figures 6 and 7). In line with CR4/CR5 being the main binding site for TERT (44), there is a significant decrease in the modification intensity of residues in P6.1 (A302, A304, G308, C313 and U314) and in adjacent junctions J6a/6.1 (A301) and J6.1/5 (U316–A318 and C320). Also, C288 becomes protected in the presence of hTERT (Figures 6B and 7C). This residue is assumed to engage in a Watson–Crick base pair with G268 at the base of P6b and this pair also forms a base triple with C267 (21) (Figure 4D). In addition, in medaka the G189 (presumably G268 in hTR) was found to contact TRBD (26), implying that the protection of C288 is caused by reduced solvent accessibility upon hTERT binding. Binding of hTERT to this region may also explain the enhanced modification of C266 in the presence of hTERT (Figure 6A and Supplementary Figures S4 and S5). Importantly, in medaka A199 (A301 in hTR) interacts via its N6 directly with the TRBD, while P6.1 uses its backbone moieties to contact the medaka TRBD (26). Variants of A301 drastically reduced telomerase activity (Supplementary Figure S3) suggesting that A301–TRBD interaction is critical for telomerase function in hTR as well. A very interesting finding was the strong modification of G308 in L6.1 in the absence of hTERT, but this residue becomes fully protected in its presence, while the modification intensity of the neighbouring U307 remains unchanged (Figure 6B and Supplementary Figures S4A and S5B). As in the G308A mutant neither hTERT binding nor telomerase activity is reduced (45), the protection of G308 in presence of hTERT suggests that the protein changes the local environment, precluding the keto-enol tautomer form of G308 and in turn its methylation. Alternatively, it remains possible that G308 contacts hTERT directly, but the interaction is not essential for complex formation, or a novel tertiary contact is formed within hTR.

The pseudoknot/template domain of hTR is the second binding site of hTERT (14). The DMS modification pattern of hTR in the presence/absence of hTERT is similar indicating that *in vivo* the P2b/P3 pseudoknot and its triple helical scaffold are formed independent of hTERT (Figure 7B and Supplementary Figure S6). Only within the extension of the pseudoknot distinct conformational changes were detected. In the P2a.1 U68 is more protected in the presence of hTERT (Figure 7B and Supplementary Figures S4C and S5A). This effect might indicate a direct contact with hTERT or is the consequence of structural stabilization of hTR upon hTERT binding. The latter is supported by the fact that the two residues C75 and C88 that are positioned in the interhelical junctions J2a.1/2a and J2a/2b are more accessible to DMS in the presence of hTERT (Figure 7B). In addition, we observed that in the presence of hTERT the modification of nucleotides in and downstream of the template (A48–C52, A59, A61, A62) is enhanced (Figure 7A,C and Supplementary Figures S4C and S5A). This suggests that hTERT induces a specific geometry of the template region, making it more exposed for interaction with the telomere and the incoming dNTP for telomere extension. Despite the observed enhancements in the template region, this element remains rather buried within the RNP, as most of the residues in the template are only weakly to moderately modified by DMS (except for some uridines).

As expected, hTERT did not induce structural changes within the H/ACA scaRNA domain of hTR (Supplementary Figure S7). Overall, our findings correlate well with the observation that TERT has a higher affinity for CR4/CR5 domain than for the pseudoknot/template domain (44). As we observed only very few discrete hTERT-induced structural changes within hTR, we propose that hTR forms a preorganized scaffold for binding of hTERT.

### hTR contains numerous pseudouridines

**Figure 8.**
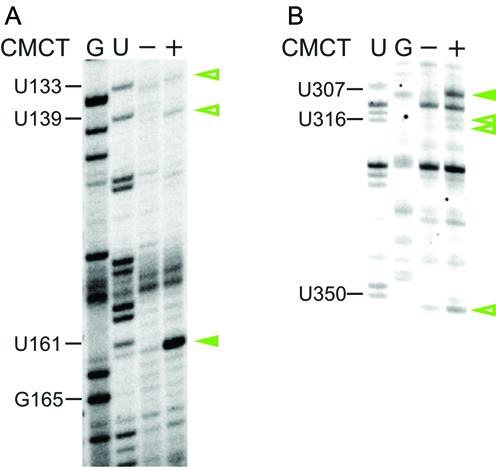
Identifying pseudouridines within hTR. Representative primer extension gels showing the CMCT modification pattern of hTR: (**A**) J2a/J2a.1, J2a.1/3 and (**B**) P6.1, J6.1/5, P4.1 and P4.2. G, U represent sequencing lanes. The – lane represents a negative control and shows natural stops encountered during reverse transcribing unmodified hTR into cDNA. + lane represents the CMCT pattern of hTR. The presence of CMCT modification at the N3 of Ψ induces termination of reverse transcription. These CMCT-induced RT stops (green arrows) are revealed by comparing the – and + lanes and excluding the natural stops. Strong modifications are highlighted with filled green arrowheads, while open green ones indicate weak modifications. The modifications were plotted onto the secondary structure map of hTR (Supplementary Figure S9).

Intrigued by the fact that 19 Us were uncommonly reactive to DMS *in vivo* (Figure 3 and Supplementary Figure S9) and 7 of these (U47, U53, U306, U307, U314, U316, U416) are highly conserved among vertebrates, we assessed whether these nucleotides are also subject to posttranscriptional modification. Notably, Kim *et al*. (22) detected six pseudouridines (Ψ159, Ψ161, Ψ179, Ψ306, Ψ307 and Ψ316) in hTR, four of which were also methylated by DMS (Ψ161, Ψ306, Ψ307 and Ψ316; Supplementary Figure S9). Since we overexpressed hTR in HEK293 cells, it was necessary to re-examine sites of pseudouridylation and to compare these Ψ positions with the Us modified by DMS (Figure 8 and Supplementary Figures S8 and S9). By using CMCT probing (35), we identified a total of 18 Ψ in hTR and 14 of these correlated with DMS modification sites. Interestingly, there is also a good agreement between the modification intensities observed for DMS and for CMCT, which is used to detect pseudouridines, as both probes target the N3 atom. For example, U161 and U307 displayed a strong modification in both cases, while residues U314 and U316 were weakly modified both by DMS or CMCT (Figure 8 and Supplementary Figure S9). Given that most uracils, which were methylated by DMS, turned out to be pseudouridines, it seems that these are more reactive to DMS that uracil. Some residues are identified as Ψ (U38, U139, U179 and U358) but are not reactive to DMS (Figure 8 and Supplementary Figures S8 and S9). Given the fact that the DMS modifications reflect the intracellular structure of hTR and the Ψ are identified in the denatured hTR extracted from HEK cells, this implies that in the native, folded structure the N3 of those Ψs are hindered for DMS modification. Other residues (U68, U83 and U306) were reactive to DMS but could not be assigned for Ψ due to reverse-transcription stops or poor signal-to-noise ratio at these positions (Figure 8 and Supplementary Figure S8B). Most importantly, these data confirmed that overexpressed hTR was indeed correctly processed in the cell and allowed detecting additional Ψs in hTR.

## DISCUSSION

Here we determined the intracellular structure of hTR and its interaction with hTERT. Most importantly, the pseudoknot with its triple helix forms stably *in vivo* independent of hTERT. Previously, *in vitro* FRET biophysical experiments suggested that the pseudoknot could only form when hTR is complexed with hTERT to form a catalytically active telomerase (46). Similar experiments were reported for the Tetrahymena telomerase (47). However, in contrast to our data, the FRET analysis indicated that a stably folded pseudoknot could only form in the isolated state, and not in the context of full-length Tetrahymena telomerase RNA. The main difference from these *in vitro* studies and our data is that we determined the structure of the telomerase RNA in its natural environment inside the cell, where a great diversity of factors potentially influence RNA folding and these conditions differ from the artificial, *in vitro* folding conditions. Earlier DMS probing of hTR in HeLa nuclei extracts revealed that a stable pseudoknot does not exist *in organello*, suggesting that this long-range tertiary interaction is formed only temporarily *in vivo* (48). In contrast to this study, we applied the DMS directly onto the living HEK cells, and not onto nuclei extracts, therefore one explanation for the observed difference in the two structures might come from different technical set-ups. Our finding is further supported by the fact that disease-related mutations in the conserved P2b/P3 element disrupt the base triples and destabilize the pseudoknot *in vivo* (Zemora and Waldsich, unpublished data). The modification pattern of the nucleotides within the extended pseudoknot indicated that these hold interesting structural information to be discovered. For example, the intricate DMS pattern of J2a.1/3 implies that these residues are involved in tertiary contacts shaping this large joining segment. Although the template region is located within a long single-stranded junction, it is conceivable that yet unknown tertiary interactions may bury the template within hTR as well as in the hTR–hTERT complex, resulting in reduced accessibility to DMS. In brief, it appears that hTERT has only limited influence on the pseudoknot and its extensions, but induces a conformational change leading to a exposure of the template region, a crucial step in DNA/RNA hybrid formation and subsequently telomere elongation. As the conserved CR4 and CR5 are part of a three-way junction, the associated junctions (J5/6a, J6a/6.1 and J6.1/5) are likely to play a crucial role for the structural organization of this domain. The observed *in vivo* modification pattern suggests that the respective residues form an intricate H-bonding network, which in turn may facilitate stacking of P6a on P5 with P6.1 pointing away from this pseudocontinuous helix, as observed for the medaka TR (26).

The main binding site of TERT is the conserved CR4/CR5 element (44). In line with this, we observed most of the hTERT-induced structural changes in this domain. Parallels can be drawn between the reduced accessibility of the residues in this region and the crystal structure of the medaka CR4/CR5–TRBD (26). First, the non-canonical base-pair A301•G315 is formed *in vivo* in hTR and is important for the telomerase functionality. Secondly, the medaka P6.1 interacts with TRBD via its backbone, which might explain the protection of A302, A304, C313 and U314 residues in the P6.1 helix in the presence of hTERT. Third, TRBD makes additional contacts with L6 of the medaka CR4/CR5 domain (26), which corresponds to the internal loop separating P6a and P6b in hTR. This interaction could be responsible for the observed conformational changes therein (enhancement of C266, protection of C288). Whether these changes are due to a direct contact between hTR and hTERT or to a specific conformation induced by hTERT binding to a nearby structural element remains to be analysed. Furthermore, in medaka telomerase L6.1 was found to be proximal to CTE (26), which might explain the total protection observed for G308 in the presence of hTERT. Even though this residue is dispensable for hTERT binding and for catalysis (45), its interaction with the CTE may stabilize the telomerase holoenzyme *in vivo*.

Measurements of the number of hTR and hTERT molecules in HEK and HeLa cells indicated that hTR is in excess of hTERT and each of these two components might have a number of molecules that are not assembled into active telomerase (49). Here, we provide clear evidence that in our transfection system these two components interact *in vivo*, and that the majority of hTR is in complex with hTERT. The most reasonable example is given by the total protection of residue G308 in the presence of hTERT (Figure 6B). The fact that this residue is modified in the absence of the protein, but becomes fully protected in the presence of hTERT indicates that the majority of the hTR molecules are in the complex with the protein. If only a small population were in the complex, we would have observed moderate modifications at this residue in the presence of hTERT and not a full protection. Moreover, we observed a strong protection (≈80%–90%) of residue C288 and the total protection of C313 (Figure 6B and Supplementary Figures S4 and S5), which further support the idea of efficient complex formation. However, even if the protein binds the high-affinity CR4/CR5 domain, the dynamical part of hTR, namely the pseudoknot might not be in a homogenous conformation. This might explain the few DMS modifications observed in the extended pseudoknot region in the presence of hTERT. Alternatively, these discrete modifications could be the result of the technical limitations of the DMS probing method, as DMS reactivity is limited to A and C residues. In this case, it might be that most of hTR–hTERT contacts in this domain do not involve the Watson–Crick edge of the A and C residues, or that the interactions are weak and therefore difficult to detect. Therefore, it is most probable that hTERT-induced conformational changes are beyond the observed ones. Applying additional techniques like UV cross-linking or/and *in vivo* SHAPE analysis would provide additional information about hTERT/hTR interaction.

In comparison with the crystal structure of an H/ACA hairpin in complex with archaeal snoRNA-binding proteins (50), P7, the 3′ pocket and P8a may interact with Dyskerin, while Nop10 would be expected to make backbone contacts with P8a. Most interestingly, L7ae (Nhp2 in human) was found to bind to a terminal k-turn motif (50). In this regard, binding of Nhp2 may also depend on a structural element harbouring non-canonical base pairs. Indeed, even though the Watson–Crick base pairs of P8a are supported by phylogeny (8), we observed that the respective As and Cs are modified by DMS, implying that these residues form an irregular helix. As only the 3′ pocket, but not the 5′ pocket was found to be important for hTR accumulation (51), the special conformation of the P8a might be important for hTR biogenesis.

In line with the DMS-modified uracil positions, also most of the pseudouridines are located in structurally important region, such as the extended pseudoknot, the template and P6.1. Since the isomerization of uracil to pseudouridine results in an NH-donor at the Hoogsteen face of Ψ, this enables tertiary contacts that uracil cannot form, thereby stabilizing RNA structure. Determining the role of these modified bases would bring more understanding of the telomerase architecture and mechanism *in vivo*. In P6.1 we detected U314 to be reactive to CMCT, therefore implying that this conserved residue is a pseudouridine. Interestingly, in the medaka CR4/CR5–TRBD crystal the counterpart of U314 is close to sequence-specific contacts (26). U212 (U314 in hTR) base pairs with A200 (A302 in hTR) and is thus adjacent to A199 (A301 in hTR), which contacts the TRBD via its N6 atom. At the same time, the backbone of U212 (U314 in hTR) and neighbouring residues was found to interact with TRBD (26). In hTR, the presence of Ψ314 at this position might therefore stabilize the CR4/CR5–TRBD assembly *in vivo*.

Our experiments clearly show that *in vivo* hTR forms a pre-organized scaffold for the assembly of the catalytic subunit hTERT. The most significant changes are in the CR4/CR5 domain and in the template region. The most intriguing finding is that hTERT exposes the template region of hTR *in vivo*, thus making it accessible for telomere elongation.

## SUPPLEMENTARY DATA

Supplementary Data are available at NAR Online.

SUPPLEMENTARY DATA
