# Supplementary material for: Human telomerase reverse transcriptase binds to a pre-organized hTR in vivo exposing its template
Source: Nucleic Acids Res. 2015 Oct 19;44(1):413–25. doi: 10.1093/nar/gkv1065 (PMC4705647; doi:10.1093/nar/gkv1065)
Supplement: SUPPLEMENTARY DATA [file supp_44_1_413__index.html]

Human telomerase reverse transcriptase binds to a pre-organized hTR in vivo exposing its template — SUPPLEMENTARY DATA 

# Human telomerase reverse transcriptase binds to a pre-organized hTR *in vivo* exposing its template

## SUPPLEMENTARY DATA

- SUPPLEMENTARY DATA
