# Supplementary material for: Human telomerase reverse transcriptase binds to a pre-organized hTR in vivo exposing its template
Source: Nucleic Acids Res. 2015 Oct 19;44(1):413–25. doi: 10.1093/nar/gkv1065 (PMC4705647; doi:10.1093/nar/gkv1065)
Supplement: SUPPLEMENTARY DATA [file supp_gkv1065_nar-01952-f-2015-File010.pdf]

Supplementary Information

Human telomerase reverse transcriptase binds to a pre-organized hTR *in vivo* exposing its template

Georgeta Zemora[1,]*, Stefan Handl[1] and Christina Waldsich[1]

[1]Department of Biochemistry and Cell Biology, Max F. Perutz Laboratories, University of Vienna, Dr. Bohrgasse 9/5, A-1030 Vienna, Austria

*To whom the correspondence should be addressed. Tel: + 43 1 4277 52805; Fax: + 43 1 4277 9528; Email: georgeta.zemora@univie.ac.at

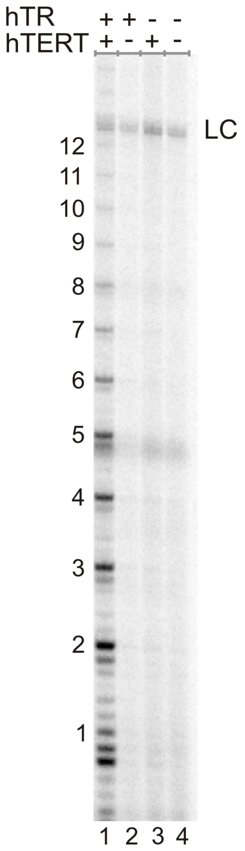

Supplementary Figure S1. Direct telomerase assay with recombinant telomerase reconstituted *in vivo.* Telomerase activity in HEK extracts was determined by direct elongation of a telomeric primer $(TTAGGG)_3$ in the presence of dATP, dTTP and ^{32}P-dGTP. When both hTERT and hTR are co-transfected (lane 1), the reconstituted telomerase is catalytically active and extends the telomeric oligo by several repeats, denoted as +1, +2, + etc. to the left of the gel. The telomeric extensions could not be extended either in the absence of the overexpressed hTERT (lane 2) or hTR (lane 3), or with endogenous HEK extracts (lane 4). LC, loading control.

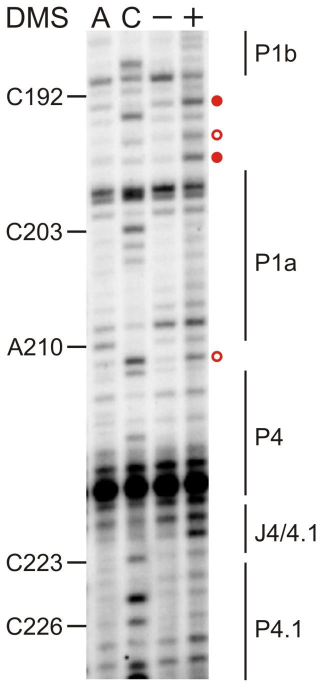

Supplementary Figure S2. Mapping the intracellular structure of the P1b, P1a and the 5' strand of the hypervariable region. Representative primer extension gel showing the *in vivo* DMS modification pattern of the P1b (3' strand), P1a (3' strand), P4 (5' strand), P4.1 (5' strand) and the 5' strand of the J4/4.1 junction. Lanes and the symbol code are designated as in Figure 2. The high GC content of this region impedes obtaining high-resolution gels, resulting in frequent pausing of reverse transcriptase during primer extension and in turn strong natural stops, as observed on the gel.

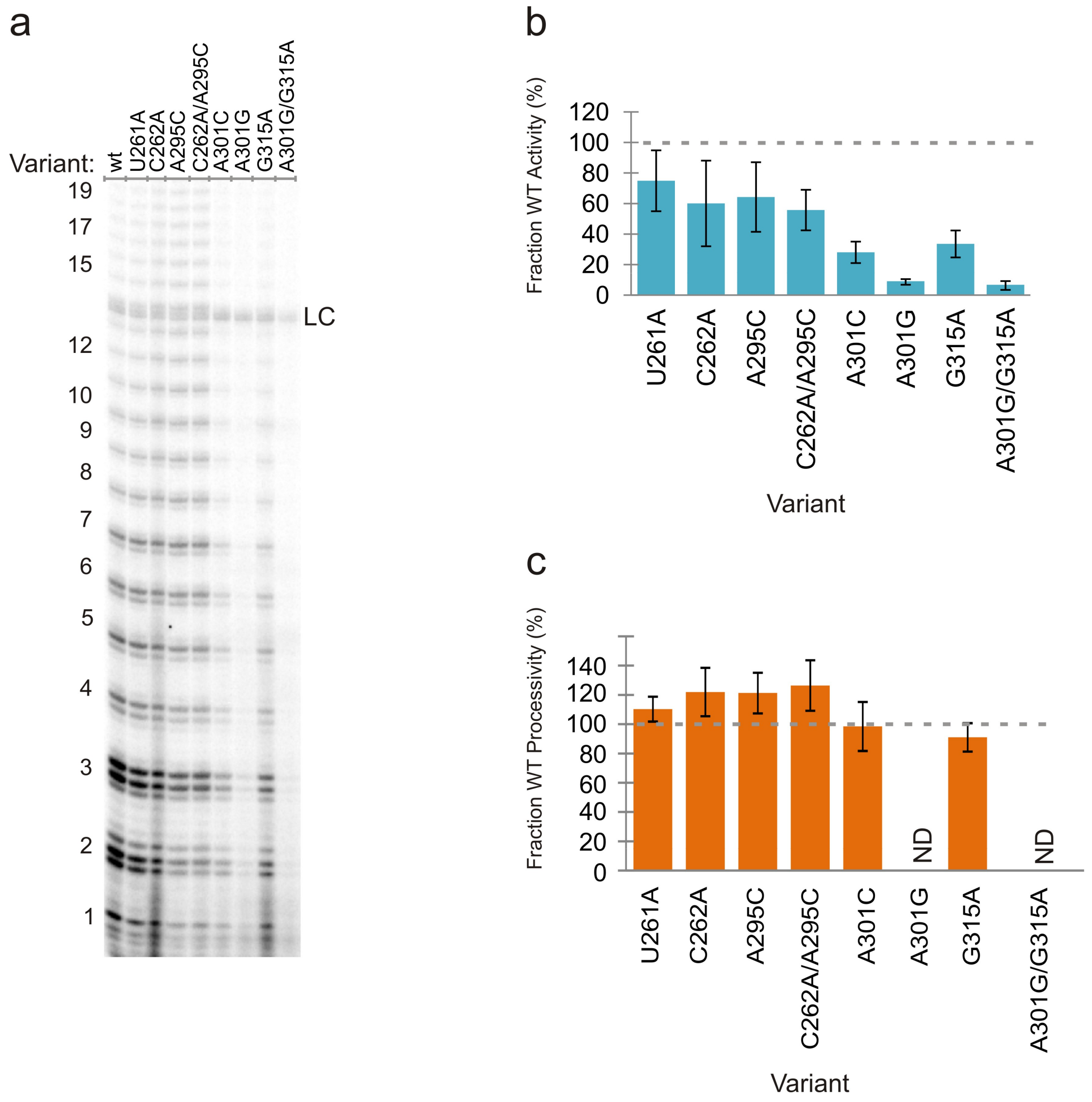

Supplementary Figure S3. Analysis of structural elements within the CR4/CR5 domain.

(a) Direct telomerase assay in cell extracts of hTR variants reconstituted in HEK cells. LC denotes a loading control. **(b)** Comparison of relative activities of mutant telomerase relative to WT. **(c)** Comparison of relative processivity of mutant telomerase relative to WT.

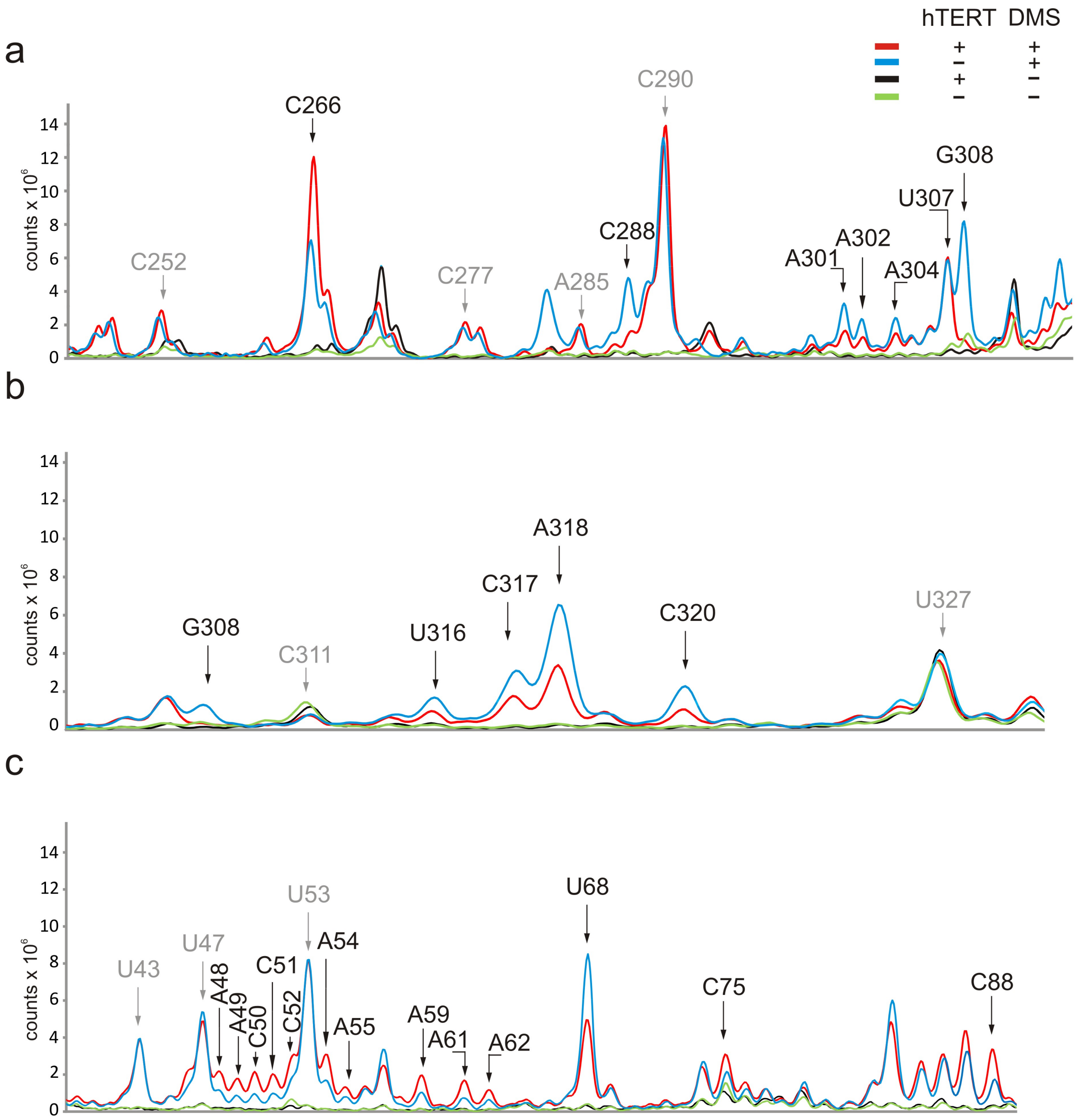

Supplementary Figure S4. Structural alterations in the CR4/CR5 domain, template region and extended pseudoknot upon hTERT binding. The normalized plot describes the modification intensity of nucleotides shown in the gels of (**a-b**) Figure 6; (**c**) Figure 7a,b in the presence of hTERT (red) and in the absence of hTERT (blue). The respective RT controls are shown in black and green, respectively. Nucleotides whose modification intensity is changed due to the presence of hTERT are labelled in black. In contrast, nucleotides that remain equally modified in the presence or absence of hTERT are given in grey and were therefore used for normalization.

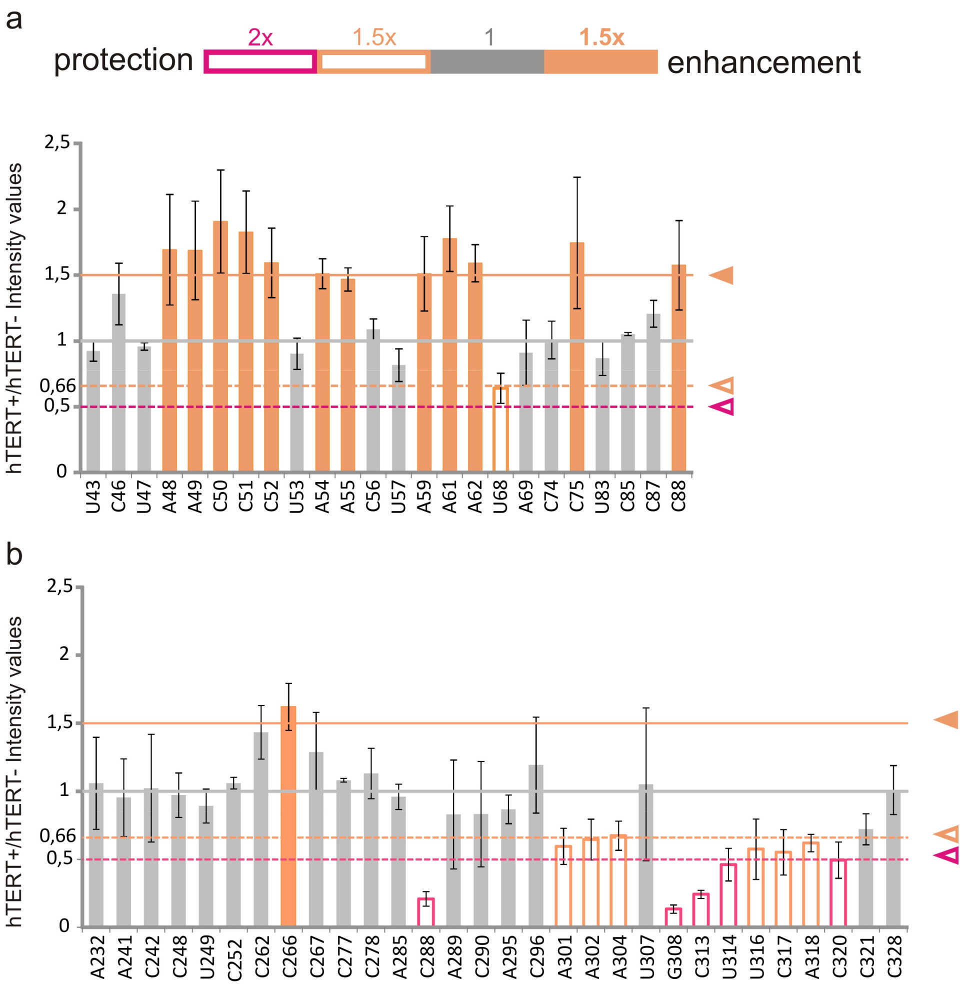

Supplementary Figure S5. The DMS modification intensity of the nucleotides in the template, extended pseudoknot and CR4/CR5 domain changes in the presence of hTERT

The normalized plots display the modification intensity of nucleotides shown in the (**a**) template region (gel in Figure 7a), pseudoknot domain (gel in Figure 7b) and (**b**) CR4/CR5 domain (gels in Figure 6). The average band intensities from lane hTERT- (no hTERT) were calculated and the mean value was used to normalize bands in the lane hTERT+ (hTERT is present). As seen, a value of 1 represents equal modification intensity in the absence or presence of hTERT. In the presence of hTERT, intensity values that are 1.5 fold higher are considered as enhancement (filled orange bars), and those 1.5 fold (0.66) or 2 fold lower (0.5) were considered as protections (open orange bars, open magenta bars respectively). Nucleotides whose modification intensity remains unchanged in hTERT presence are shown as gray bars. The histograms and standard deviations were calculated from at least three independent experiments. The arrows are designated as in Figure 7c.

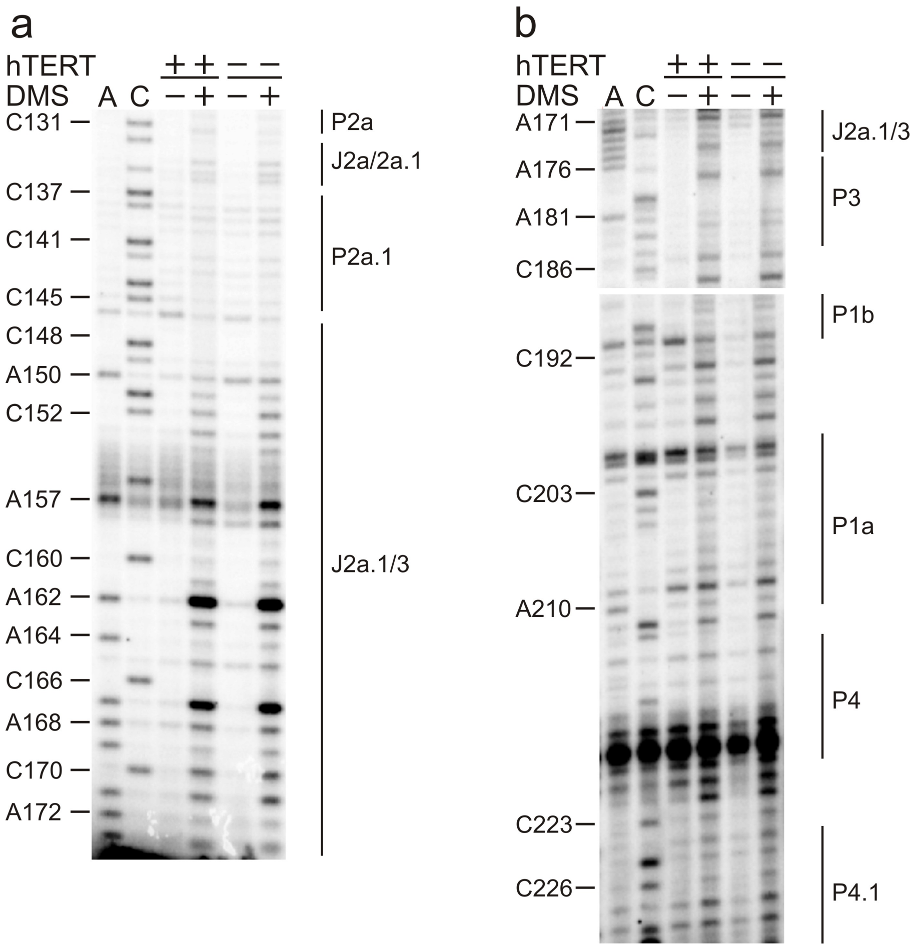

Supplementary Figure S6. Formation of the pseudoknot is independent of hTERT.

Representative gels showing the modification intensity of the nucleotides in the (**a**) stems P2a.1, P2a and their joining segments as well as of (**b**) P3 (3’ strand), P1b (3’ strand), P1a (3’ strand), P4 (5’ strand) and P4.1 (5’ strand) in the presence (hTERT+) and absence (hTERT-) of hTERT. Lanes are designated as in Figure 6.

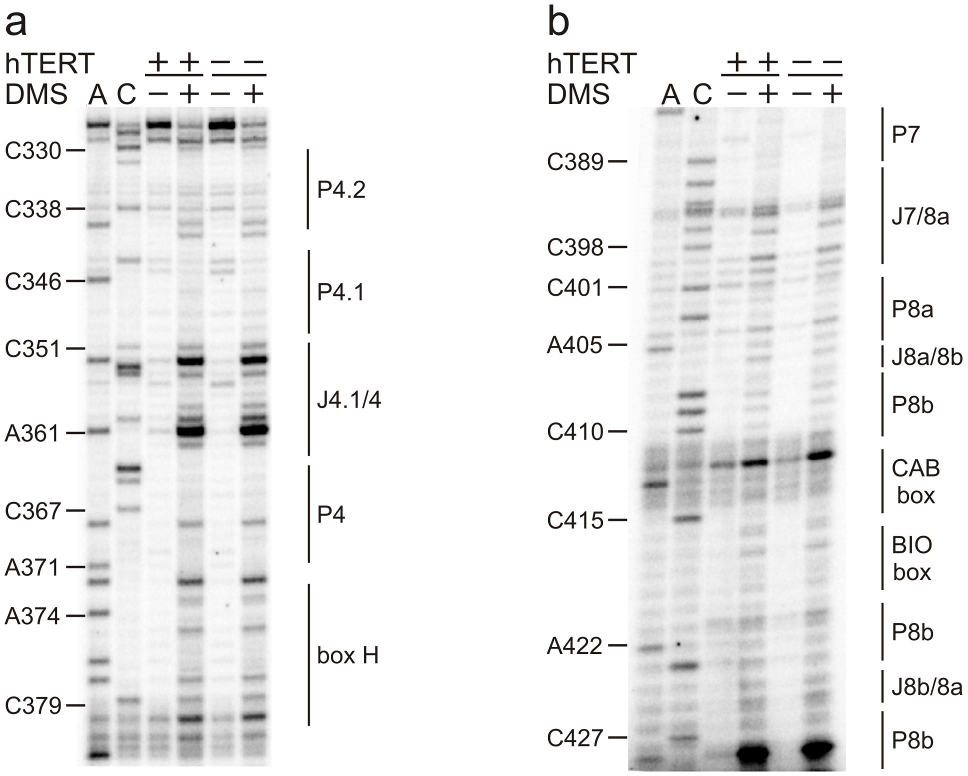

Supplementary Figure S7. hTERT does not influence the architecture of the H/ACA scaRNA domain. Representative primer extension gels showing the modification intensity of the nucleotides in the (**a**) 5' and (**b**) 3' H/ACA hairpin in the presence (hTERT+) and absence (hTERT-) of hTERT. Lanes are designated as in Figure 6.

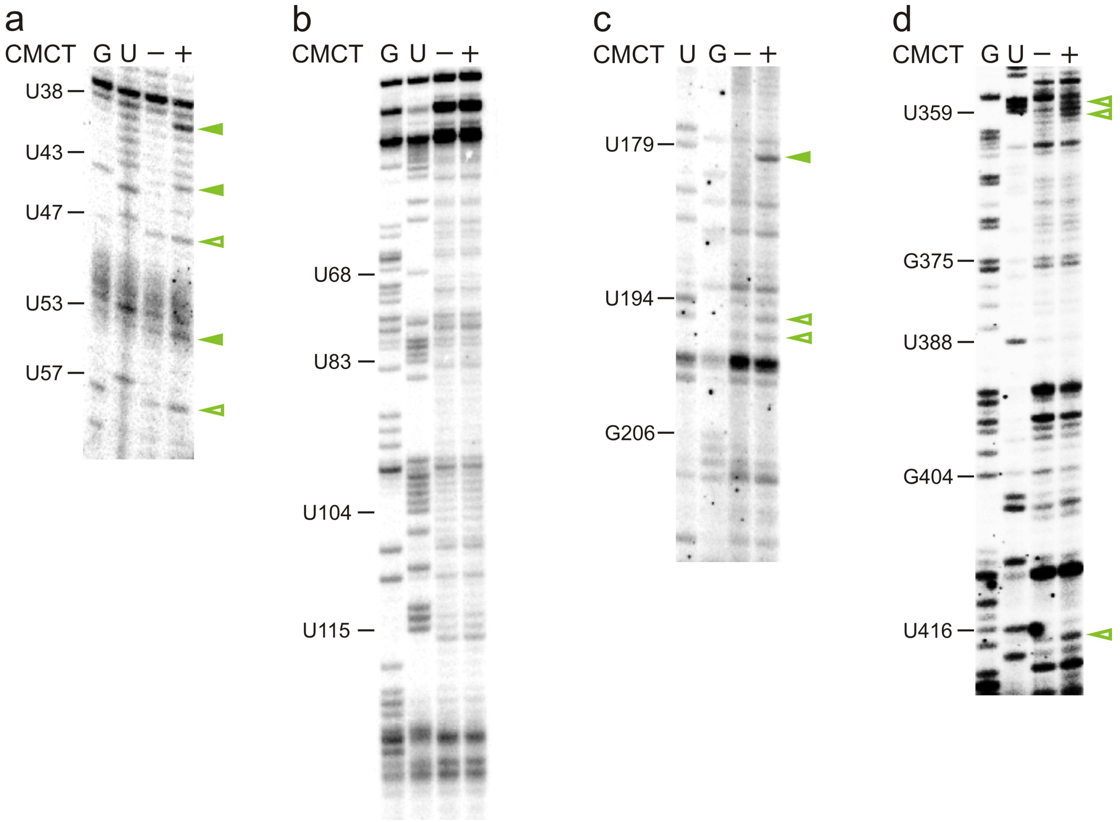

Supplementary Figure S8. Identifying pseudouridines within hTR. Representative primer extension gels showing the CMCT modification pattern of hTR: (**a**) template region; (**b**) P2a.1, J2a.1/2a, P2a, J2a/2b; (**c**) P3, P1b, P1a; (**d**) H/ACA scaRNA domain (except nts 419-451). Lanes and the symbol code are designated as in Figure 8. The modifications were plotted onto the secondary structure map of hTR (Supplementary Figure S9).

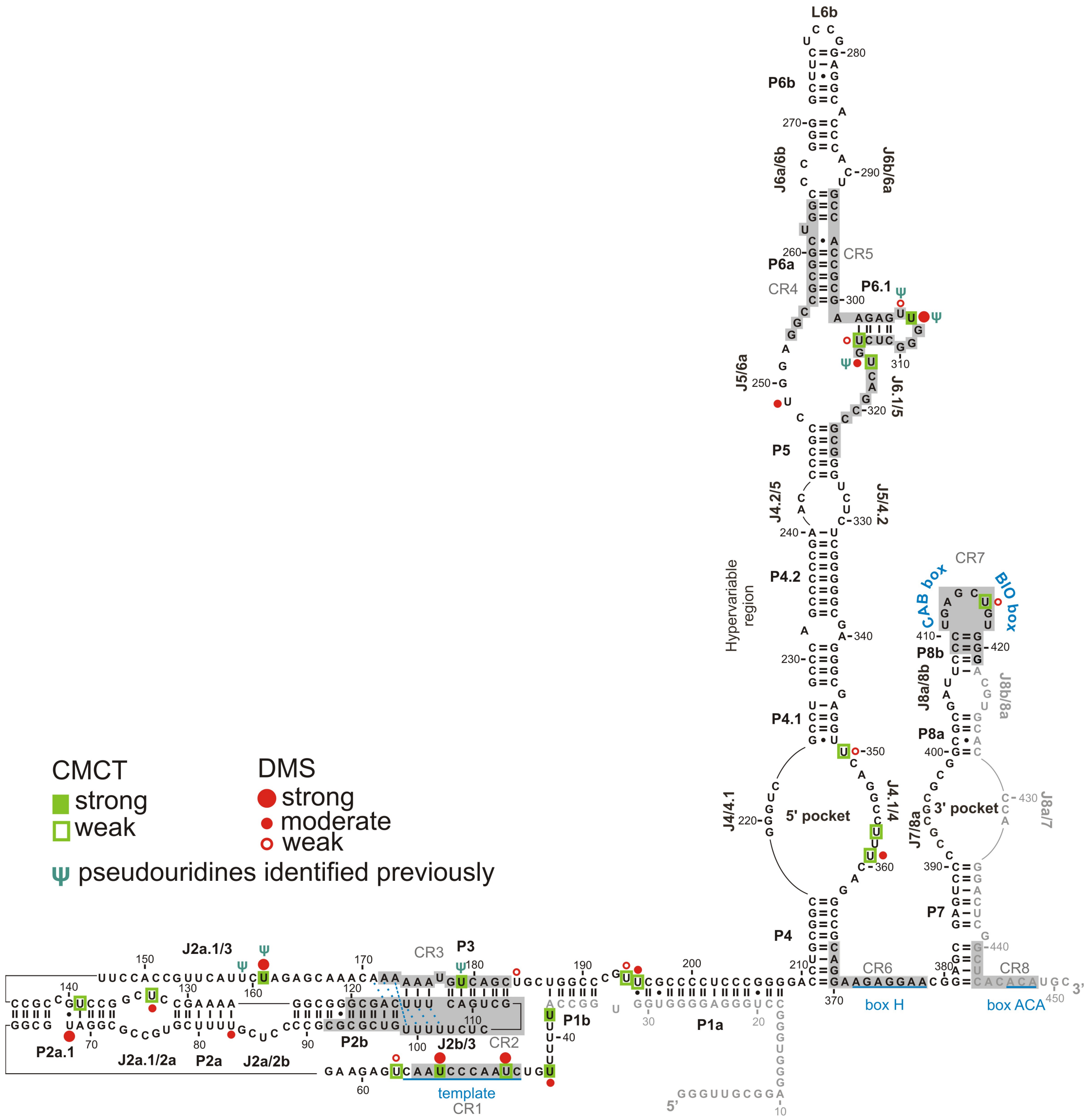

Supplementary Figure S9. Sites of pseudouridylation coincide to great extent with methylation by DMS. Summary map (of three individual experiments), in which uracil residues accessible to DMS are indicated with red circles, while pseudouridines identified by CMCT modification are indicated with green squares. Previously identified pseudouridines (1) are marked with a green symbol. Red open circles indicate residues with weak reactivity of U to DMS, while red filled circles represent strongly (large circles) or moderately (small circles) modified nucleotides. Green filled squares represent U residues that are strongly modified by CMCT, while open squares represent residues that are weakly reactive to CMCT. Residues coloured in light grey (nts 1-38 and 422-450) could not be mapped by reverse transcription, as the very 3' end served as primer binding site and the nucleotides at the very 5' end were not well resolved on the denaturing gel. Average values were calculated from at least three independent experiments.

Supplementary references

1. Kim, N.K., Theimer, C.A., Mitchell, J.R., Collins, K. and Feigon, J. (2010) Effect of pseudouridylation on the structure and activity of the catalytically essential P6.1 hairpin in human telomerase RNA. *Nucleic acids research*, **38**, 6746-6756.
